# Evaluation of alveolar bone preservation and oxidative stress reduction with açai in Wistar rats with induced apical periodontitis

**DOI:** 10.1111/iej.14247

**Published:** 2025-05-08

**Authors:** João Daniel Mendonça de Moura, Vinicius Ruan Neves Dos Santos, Leonardo Oliveira Bittencourt, Fabrício Mezzomo Collares, Paulo Fernando Santos Mendes, José Mario Matos‐Sousa, Beatriz Rodrigues Risuenho Penaido, José Messias Perdigão, Herve Rogez, Rafael Rodrigues Lima, Patricia de Almeida Rodrigues

**Affiliations:** ^1^ Department of Dentistry, School of Dentistry Federal University of Pará Belém Pará Brazil; ^2^ Laboratory of Functional and Structural Biology, Institute of Biological Sciences Federal University of Pará Belém Pará Brazil; ^3^ Dental Materials Laboratory, Department of Conservative Dentistry, School of Dentistry Federal University of Rio Grande do Sul Porto Alegre Rio Grande do Sul Brazil; ^4^ Center for Valorization of Amazonian Bioactive Compounds, College of Biotechnology Federal University of Pará Belém Pará Brazil

**Keywords:** alveolar bone preservation, apical periodontitis, clarified açai, *Euterpe oleracea* Martius palm, micro‐CT analysis, oxidative stress reduction

## Abstract

**Aim:**

This study aimed to investigate the effects of açai on bone and systemic damage caused by apical periodontitis (AP) in an animal model.

**Methodology:**

In this experimental study, 32 male Wistar rats were randomly allocated into six groups—Control 14 days (*n* = 4), Control 28 days (*n* = 4), AP 14 days (*n* = 4), AP 28 days (*n* = 4), AP plus açai treatment for 14 days (*n* = 8) and AP plus açai treatment for 28 days (*n* = 8). Apical periodontitis was induced under general anaesthesia by exposing the pulp of the first molars to the oral environment. Daily treatments were administered by gavage at a dose of 0.01 mL/g, using either saline solution or clarified açai. At the end of each experimental period, periapical lesions were quantitatively evaluated by micro‐computed tomography (micro‐CT) and histopathological analyses, whilst systemic oxidative stress was assessed through biochemical assays. Data normality was verified using the Shapiro–Wilk test, followed by one‐way anova and Tukey's post hoc test (*p* < .05).

**Results:**

Micro‐CT analysis revealed that açai reduced apical periodontitis lesion volume and improved bone quality (*p* < .05). Histopathological evaluation corroborated these findings, revealing moderate inflammation at 14 days and more pronounced, heterogeneous inflammatory responses at 28 days, with no significant differences between groups. Additionally, açai modulated systemic oxidative biochemistry, enhanced antioxidant defences and reduced pro‐oxidant damage after 28 days.

**Conclusions:**

Oral açai administration was associated with reduced progression of apical periodontitis and improved bone quality, suggesting its potential as a protective antioxidant in endodontic treatment, minimizing both local and systemic damage.

## INTRODUCTION

Apical periodontitis (AP) is caused by the entry of microorganisms into the root canals, leading to pulp necrosis (Karamifar et al., 2020). The infection spreads in the apical direction until it reaches the periradicular tissues, resulting in inflammation (Karamifar et al., 2020). AP develops in response to a contaminated root canal, resulting in significant damage to the periradicular tissue, often leading to bone resorption, which may occur with or without pus formation (i.e., an abscess) (Siqueira & Rôças, 2022). It is estimated that half of the world population has at least one tooth affected by AP (Jakovljevic et al., 2020; Tibúrcio‐Machado et al., 2021), highlighting the importance of investigating the disease, its manifestations and potential prevention strategies.

Oxidative stress plays a central role in the pathogenesis of AP (Hernández‐Ríos et al., 2017). Inflammatory mediators are activated by reactive oxygen species (ROS), which play crucial roles in signalling within the innate immune response (Inchingolo et al., 2013). Oxidant imbalance contributes locally to the formation and progression of AP through direct molecular damage and redox signalling (Hernández‐Ríos et al., 2017). Therefore, patients with AP are exposed to oxidative stress, which poses a considerable risk to their overall health (Inchingolo et al., 2013). Recent research suggests that AP not only impacts local tissues but also exacerbates oxidative stress in the peripheral blood, contributing to systemic health deterioration (Milojevic Samanovic et al., 2021). Frazão et al. (2023) demonstrated that the progression of AP induced in rats modulates systemic levels of pro‐ and antioxidants, underscoring the broader systemic implications of this condition. Specific foods and products with antioxidant properties have been tested to minimize the damage caused by this condition. For example, resveratrol administration decreases inflammatory processes in AP and reduces periapical bone resorption (Dal‐Fabbro et al., 2021). Additionally, alpha‐lipoic acid treatment has therapeutic effects on the inhibition of periapical bone loss (Aksoy et al., 2019), whilst melatonin exerts anti‐resorptive effects on bones via its anti‐inflammatory activity (Sarıtekin et al., 2019).

**FIGURE 1:**
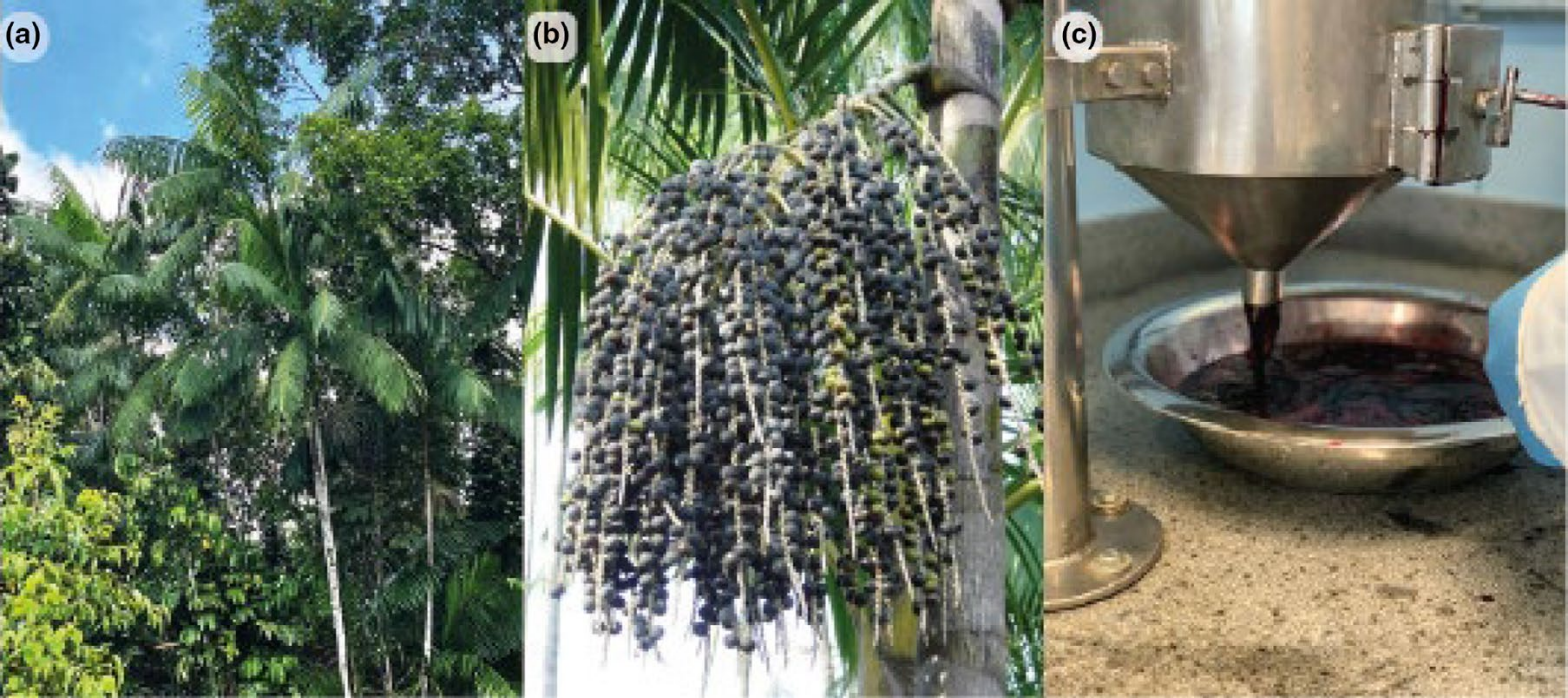
(a) The açai tree; (b) açai fruit at the stage used to prepare the pulp; and (c) processed açai ready for human consumption.

*Euterpe oleracea*, commonly known as the açai palm, is a plant native to the Amazon Basin (Figure 1a–c). Its fruit is used to prepare a juice called açai, a staple food for the local population that has been widely traded worldwide since approximately 2000. The pulp constitutes only 5%–15% of the fruit mass (Bichara & Rogez, 2011). Açai is obtained from the fruit by soaking it in lukewarm water and mechanically crushing it. The daily consumption of açai by Amazonian traditional communities is estimated to be 500 mL per 60 kg of body weight (Yamaguchi et al., 2015).

The purple colour of the fruit is due to the high concentrations of anthocyanins and phenolic compounds belonging to the flavonoid class. Phenolic compounds are the major phytochemicals in açai fruit (Earling et al., 2019). The most important polyphenols are the anthocyanins cyanidin‐3‐rutinoside and cyanidin‐3‐glycoside, followed by the non‐anthocyanin compounds homoorientin, orientin, taxifolin, deoxyhexose, vitexin and isovitexin (Dias et al., 2012, 2013). Compared to other berries, fruits or vegetables, the diverse composition and high concentration of phenolic compounds in açai pulp ensure a high antioxidant capacity, as shown either through oxygen radical absorption or total oxidant scavenging capacity measurements (Lucas et al., 2018).

Previous studies have shown that açai has an anti‐inflammatory action (de Oliveira et al., 2021), and our group recently demonstrated its effectiveness in modulating bone metabolism (de Moura et al., 2024) and minimizing the damage caused by ligature‐induced periodontitis in rats (dos Santos et al., 2022). Moreover, considering açai's potent antioxidant properties, which can be crucial in counteracting the oxidative stress underlying AP pathogenesis, this study extends such investigations to an endodontic model. In endodontics, oxidative stress, inflammatory mediators and osteoclastic activity critically influence lesion progression; thus, elucidating the protective role of açai may provide new insights for adjuvant therapies. Therefore, this study aimed to evaluate the effects of oral açai administration on bone preservation and the modulation of systemic damage triggered by AP throughout its progression at varying exposure durations. The null hypothesis tested was that oral açai administration has no effect on either bone or systemic damage.

## METHODS

### Ethics statement

**FIGURE 2:**
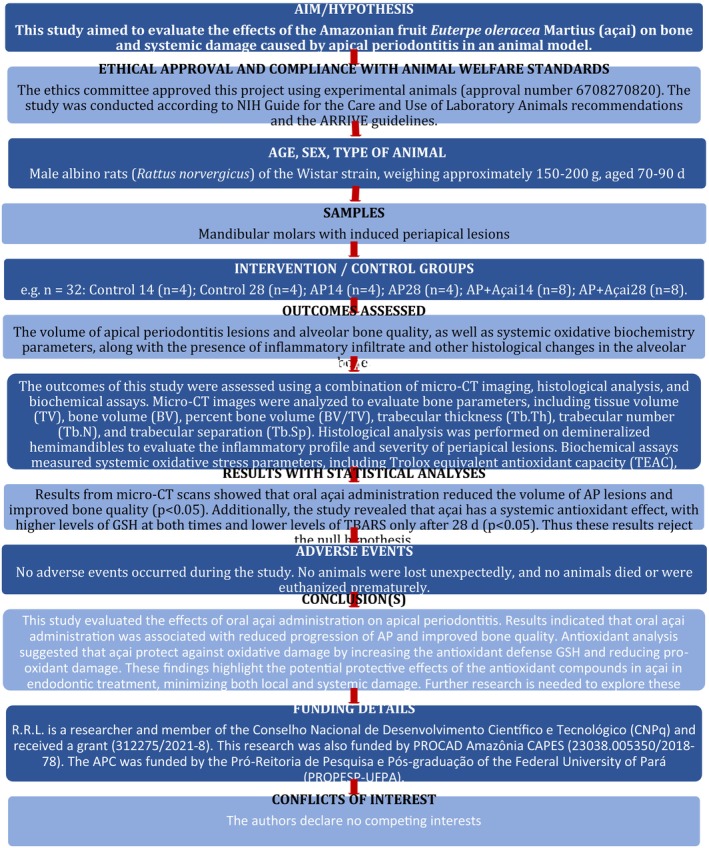
PRIASE 2021 flowchart.

This study adhered to the PRIASE 2021 guidelines (Nagendrababu et al., 2021) for reporting animal studies in endodontology, including the PRIASE flowchart (Figure 2), to ensure methodological rigour, reproducibility and transparency. The Ethics Committee approved this project involving experimental animals (approval number: 6708270820). The study was conducted in accordance with the National Institutes of Health Guide for the Care and Use of Laboratory Animals and the ARRIVE guidelines (Percie du Sert et al., 2020).

### Animals

**FIGURE 3:**
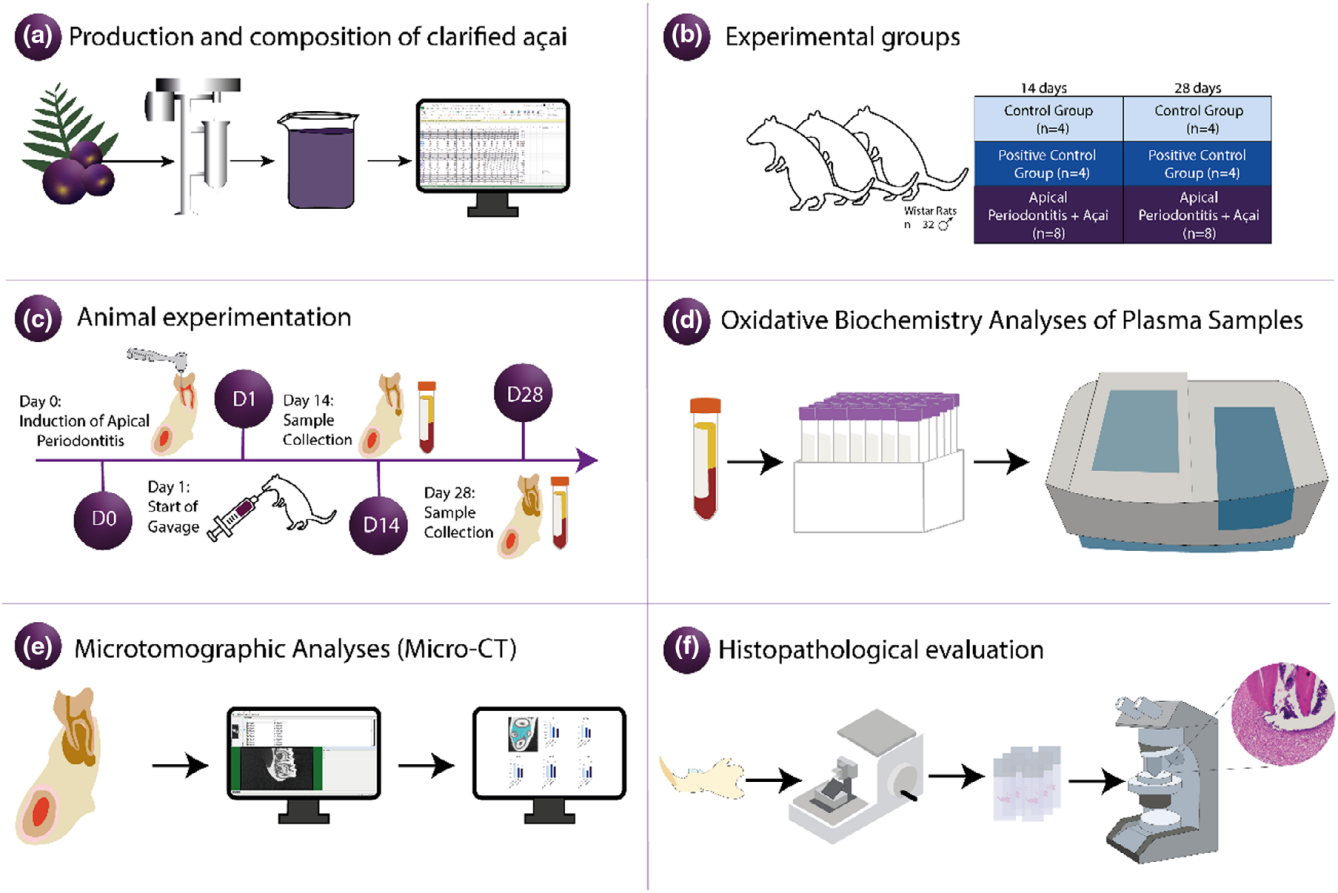
Flowchart of the experiment. (a) The production stage and measurement of the composition of clarified açai; (b) allocation to experimental groups; (c) experimental stages of the collection of the samples after 14 or 28 days; (d) evaluation of plasma oxidative stress; (e) microtomographic analysis (micro‐CT); (f) histopathological evaluation.

Thirty‐two male albino rats (*Rattus norvergicus*) of the Wistar strain weighing approximately 150–200 g (aged 70–90 days) were obtained from the central vivarium and acclimated to the vivarium of the laboratory. This species has been previously used in animal studies using the AP model proposed in this study (Hong et al., 2004; Kawashima et al., 1996, 2007). The animals were housed in polypropylene cages with ad libitum access to food and distilled water in a climate‐controlled room with a 12‐h light/dark cycle. The animal caretakers comprised master's students and undergraduate research assistants—each possessing extensive experience in animal care—as well as a technical employee of the university responsible for the animal facility. The rats were randomly divided into six groups: Control 14 (Control Group; *n* = 4; not exposed to any procedure and receiving an oral daily dose of 0.01 mL/g saline solution for 14 days); Control 28 (Control Group; *n* = 4; not exposed to any procedure and receiving an oral daily dose of 0.01 mL/g saline solution for 28 days); AP14 (Positive Control group; *n* = 4; AP induced and receiving an oral daily dose of 0.01 mL/g saline solution for 14 days); AP28 (Positive Control group; *n* = 4; AP induced and receiving an oral daily dose of 0.01 mL/g saline solution for 28 days); AP + Açai14 (Apical Periodontitis + Açai; *n* = 8; AP induced and receiving an oral daily dose of 0.01 mL/g clarified açai for 14 days); AP + Açai28 (Apical Periodontitis + Açai; *n* = 8; AP induced and receiving an oral daily dose of 0.01 mL/g clarified açai for 28 days). The sample description and the experimental steps are detailed in Figure 3.

The primary outcomes assessed include the quantification of periapical lesion volume and evaluation of alveolar bone quality. These measures are derived from micro‐computed tomography (micro‐CT) analysis. The secondary outcomes focus on systemic oxidative stress and histopathological assessments.

### Induction of apical periodontitis

Animals in the AP14, AP28, AP + Açai14 and AP + Açai28 groups (*n* = 24) underwent AP induction. The animals were anaesthetized by intraperitoneal injection of 2% xylazine hydrochloride (2 mg/mL; Syntec, São Paulo, Brazil) and 10% ketamine hydrochloride (10 mg/mL; Syntec) at doses of 9 and 90 mg/kg, respectively. After general anaesthesia, the animals were placed on an operating table that kept their mouths open, facilitating access to the posterior teeth of the mandible. The pulp of the left and right first molars was exposed using a #1/4 carbide bur for low rotation (KaVo Dental, Biberach an der Riß, Germany) coupled to an X‐Smart Plus motor (Dentsply Maillefer, Ballaigues, Switzerland) set to a speed of 1200 rpm and a torque of 4.0 N·cm. Access to the distal fossa of the first molar was obtained at a depth corresponding to the active tip of the drill. The pulp tissue was then disrupted using a #10 C‐pilot file, followed by a #25 K file, until pulp tissue exposure was confirmed by observing bleeding from the pulp (Zivanovic et al., 2022). To induce periradicular lesions, the exposed teeth were left open in the oral environment until the end of the experimental period.

Immediately after the AP induction procedure, the animals were weighed, using gloves to avoid direct hand contact and minimize stress. Animals in all groups were weighed weekly until days 14 and 28. The animals were closely monitored throughout the experimental period for signs of distress, pain or abnormal behaviour. Observations included monitoring physical activity, feeding patterns and body weight progression. All animals maintained normal activity levels, exhibited stable feeding behaviour and showed consistent weight gain, indicating effective recovery from anaesthesia and surgical intervention. No adverse events or signs of prolonged distress were observed.

To minimize pain in animals after pulp exposure, daily doses of dipyrone (100 mg/kg; Zoetis, Parsippany, NJ, USA) were administered for 3 days. A 1‐mL syringe was filled with the dose (calculated based on weight), the animal was restrained and the application area was prepared with an alcohol‐soaked swab, tensioned to allow insertion of the hypodermic needle (30 g) at the base of the tensioned area. Aspiration was performed at the appropriate site to confirm application. The needle was repositioned if signs of bleeding were observed or in the absence of negative pressure in the syringe. Once completed, the medication was administered via continuous fluid flow.

To confirm the development of AP, radiographic and histological analyses were conducted at the end of the study. Animals that did not develop AP were to be excluded from the analysis; however, all the animals exhibited lesions.

### Clarified acai juice

The juice of *E. oleracea* fruits used in this study was prepared according to a patented process (PI 1003060‐3, 04/08/2010, Brazil). Briefly, clarified açai was prepared from fresh drupes. After cleaning the fruit, pulping was performed with the addition of 0.5 L water per kilogram of fruit. The juice was subsequently microfiltered (clarification process) and stored at −22°C until the experiments. The açai obtained was a thin, translucent, wine‐coloured liquid without lipids, proteins or fibres, but rich in phenolic compounds.

In accordance with the Brazilian biodiversity regulations and the Nagoya Protocol, the use of this genetic resource is registered with the National System for the Management of Genetic Heritage and Associated Traditional Knowledge (SisGEN). The açai used in this study was sourced from Abaetetuba, Pará, Brazil (01°43′05″ S, 48°52′57″ W). The SisGEN registration number for this study was AAAFF39, ensuring compliance with the legal, ethical and regulatory standards governing research involving Brazilian natural resources.

An aliquot of açai was characterized by its total phenolic and anthocyanin contents. Total phenolic content was determined using the Folin–Ciocalteu method (Pompeu et al., 2009). The major flavonoids were quantified using two validated ultra‐high‐performance liquid chromatography‐photodiode array (UHPLC‐PDA) methods (Dias et al., 2012). Standards including orientin, homoorientin, taxifolin, vitexin, isovitexin, cyanidin‐3‐glucoside and cyanidin‐3‐rutinoside (Extrasynthèse, Genay, France) were used.

The major phenolic compounds of açai were identified and quantified as cyanidin‐3‐glucoside (112.20 mg/L), cyanidin‐3‐rutinoside (543.30 mg/L), homoorientin (184.15 mg/L), orientin (144.81 mg/L), taxifolin deoxyhexose (13.06 mg/L), vitexin (10.57 mg/L) and isovitexin (10.18 mg/L).

Gavage was performed daily with açai or distilled water for 14 or 28 days, according to the group to which the rats were allocated. The dosage used was 0.01 mL/g, based on previous studies (de Moura et al., 2024; dos Santos et al., 2022) demonstrating antioxidant properties and action on pro‐inflammatory cytokines (Souza‐Monteiro et al., 2019; Zhou et al., 2018). Oral açai administration was commenced on day 1 immediately after AP induction.

### Blood collection

After the experimental periods of 14 and 28 days, the animals were anesthetized using an intraperitoneal injection of 9 mg/kg xylazine and 90 mg/kg ketamine. After verifying the absence of corneal reflexes, blood for oxidative stress analysis was collected using a 3 mL syringe from the right atrium of the animals, stored in tubes with EDTA K2 (Becton Dickinson, USA) and centrifuged at 3000 rpm for 10 min. Subsequently, plasma and blood cells were collected and stored in microtubes at −80°C until additional analyses of glutathione (GSH), thiobarbituric acid‐reactive substances (TBARS) and Trolox‐equivalent antioxidant capacity (TEAC). Blood was collected only from the AP14, AP28, AP + Açai14 and AP + Açai28 groups (*n* = 24).

### Perfusion

Animals that had already been subjected to anaesthesia were perfused through the left ventricle of the heart with 0.9% saline solution, 1% heparinized (5000 UI, Blau Pharmaceuticals, São Paulo, Brazil) and 4% formaldehyde (CAS no. 50‐00‐0, Neon, Brazil). Subsequently, the hemimandibles were removed from both sides.

### Hemimandible collection

The mandibles were collected, trimmed and preserved in 4% formaldehyde (CAS no. 50‐00‐0, Neon, Brazil) solution with a liquid volume at least 30 times larger than the sample size. Specimens were prepared to measure the lesion volume using computed microtomography (micro‐CT) and histopathological examinations.

### Analysis of the collected samples

#### Blood oxidative stress analysis

##### Trolox‐equivalent antioxidant capacity (TEAC)

The TEAC was determined according to a method adapted from Miller et al. (1993) and modified by Re et al. (1999). A total of 2970 μL of the working solution of 2,2′‐azinobis‐3‐ethylbenzthiazoline, 6‐sulfonate (ABTS; CAS no. 30931‐67‐0, Sigma‐Aldrich, St. Louis, MO, USA) was placed in a cuvette and read. Next, 100‐μL aliquots of plasma samples were transferred to test tubes containing 900 μL of water. Aliquots of 30 μL were then transferred from the samples to cuvettes containing the radical and read after 5 min. The absorbance of the reaction mixture was measured at 734 nm for 5 min. The results were expressed as μM/mL.

##### Measurement of glutathione levels

GSH levels were measured using the method described by Ellman (1959) An aliquot (20 μL) of plasma was added to a tube containing distilled water (20 μL) and phosphate buffer saline (PBS)‐EDTA buffer solution pH 8.0 (3 mL) to perform the first measurement. Subsequently, 0.47 mmol 5,5‐dithiobis (2‐nitrobenzoic acid) (DTNB; CAS no. 69‐78‐3, Sigma‐Aldrich) was added to the solution and another measurement was performed after 3 min. The GSH concentration was expressed as μg/mL using GSH as a standard (CAS no. 70‐18‐8, Sigma‐Aldrich).

##### Determination of lipid peroxidation

TBARS, which are indicators of lipid peroxidation, were measured as described by Kohn and Liversedge (1944) and modified by Percário et al. (2012). Malondialdehyde (MDA) produced after lipid peroxidation reacts with thiobarbituric acid (TBA) to generate a chromophore. Briefly, 1 mL of 10 nM TBA (CAS no. 504‐17‐6, Sigma‐Aldrich) was added to 100 μL of samples after incubation for 1 h at 94°C. The samples were cooled, and 4 mL of *n*‐butanol (CAS no. 71‐36‐3, Sigma‐Aldrich) was added to each sample. The samples were homogenized and centrifuged at 2500 rpm for 10 min. The absorbance of the organic phase (3 mL) was measured at 535 nm. The TBARS concentration is expressed as nM/mL using MDA as a standard (CAS no. 100683‐54‐3, Sigma‐Aldrich).

#### Computed microtomography analysis

The imaging protocol was based on the work of Kalatzis‐Sousa et al. (2017). Non‐demineralized hemimandibles were scanned using a cone‐beam micro‐CT system (Skyscan 1172, Bruker, Kontich, Belgium). Each specimen was mounted on a rotating platform inside a device undergoing a 360° rotation at an intensity of 70 kV and 100 mA. The images were reconstructed in the software program inspeXio SMX‐90CT (Shimadzu, Kyoto, Japan) with a voxel size of 10 μm, a resolution of 1024 × 1024 pixels and a slice thickness of 14 μm.

Initially, the image orientation was adjusted using the first lower molar as a reference to obtain a standard orientation using the appropriate software (Data Viewer1, version 1.5.0; Bruker, Kontich, Belgium). In the sagittal plane, the cervical opening of the distal canal and the exit of the apical foramen were aligned along the *X* axis. In the coronal plane, the long axis of the distal root was aligned parallel to the *Z* axis. Finally, in the axial plane, the mesial and distal canals were aligned along the *Z* axis, keeping both canals parallel. The *Y* axis was not used for the orientation. Regardless of the orientation, the coronal, sagittal and axial planes were maintained perpendicular to each other. The images from the sagittal and coronal planes were exported and analysed using the CTAn software (Skyscan, Kontich, Belgium). A suitable threshold was required to distinguish bone resorption cavities from the entire specimen (Aksoy et al., 2019). Therefore, the threshold was set as follows: the lower limit was between 0 and 255 (grey values) and the upper limit was at the top end of the brightness spectrum, representing the highest bone density value.

The region of interest (ROI) was delimited using a custom function to select only the region corresponding to the periradicular lesion (sagittal sections) or periodontal ligament (control groups), and the interradicular region (coronal sections) across all sets of images. The calculated parameters were tissue volume (TV), expressed in cubic millimetres (mm^3^); bone volume (BV), expressed in cubic millimetres (mm^3^); per cent bone volume (BV/TV), expressed as a percentage (%); trabecular thickness (Tb.Th), expressed in millimetres (mm); trabecular number (Tb.N), expressed per millimetre (1/mm); and trabecular separation (Tb.Sp), expressed in millimetres (mm). RadiAnt DICOM Viewer (Version 2022.1, Medixant, Poznań, Poland) software was used to create figures to illustrate the selected ROI.

#### Histopathological evaluation

Other hemimandibles from each animal were collected and post‐fixed in 4% formaldehyde (CAS no. 50‐00‐0, Neon) for 24 h. After 90 days, the samples were demineralized in 10% EDTA (CAS no. 60‐00‐4, Sigma‐Aldrich). The pieces were then dehydrated in alcohol, diaphanized in xylene and embedded in paraplast (CAS no. 145686‐99‐3, Sigma‐Aldrich). Following inclusion, the materials were sliced on a Leica RM 2045 microtome (Leica Microsystems, Nussloch, Germany) with a thickness of 5 μm in the mesiodistal orientation and placed on individual slides (Chemelo et al., 2022). Sections were stained with Harris' haematoxylin (C.I.75290, Easypath, São Paulo, Brazil) and eosin (C.I. 45380, Imbralab, São Paulo, Brazil) and photomicrographed using a digital colour camera (CyberShot DSC W‐230; 4× optical zoom, Sony, Tokyo, Japan) attached to an optical microscope (Leica QWin Plus; Leica Microsystems, Nussloch, Germany). The inflammatory profile of the periapical lesions was determined using semi‐serial sections along the entire length of the mandible. The severity of the lesion was then determined based on the intensity, characteristics, extent of inflammatory infiltration, preservation of the cementum and integrity of the bone.

### Statistical analyses

Sample size calculation was performed based on a study by Pinto et al. (2020) using GPower 3.1.9.4 (Faul et al., 2007). The primary outcome was the intergroup difference in the mean periapical lesion volume measured by micro‐CT. Using a power of 0.80 and a significance level (*α*) of .05, an effect size of 2.56 was calculated to detect a difference of 3.39 mm^3^ between groups with a standard deviation of 1.32. Consequently, 24 animals (i.e., 4 animals per group) were required. However, accounting for a 20% loss rate commonly reported in the literature, another 8 animals were allocated to the açai groups, resulting in a final total of 32 animals. Data distribution normality was analysed using the Shapiro–Wilk test. Statistical analyses were performed using a one‐way anova followed by Tukey's test. The use of a one‐way anova without directly comparing treatment times was considered the most appropriate, as the treatment duration varied between the groups. Body weight data were evaluated using repeated‐measures two‐way anova. The *t*‐test was used for the biochemical analysis since only two groups were compared, as the analyses were conducted only on animals with lesions. All results were expressed as mean ± standard error of the mean (SEM). Statistical significance was set at *p* < .05. GraphPad Prism 9.0 software (GraphPad, San Diego, CA, USA) was used for the statistical analyses. Descriptive analysis was performed for histopathological evaluation.

## RESULTS

### Açai gavage did not affect the body weight of animals

The animals were weighed weekly, and all groups gained weight (*p* < .0001). At the end of the experimental protocol, no significant difference was observed in the body weight between the control group and the açai groups for the same period (Control 14: 194.75 ± 18.06; AP14: 196.25 ± 9.17; AP + Açai14: 187.25 ± 13.87; Control 28: 207.25 ± 12.68; AP28: 202.25 ± 22.33; AP + Açai28: 202.62 ± 26.06; *p* > .05).

### Micro‐computed tomography analysis

#### Açaí ingestion was associated with reduced periapical bone loss

**FIGURE 4:**
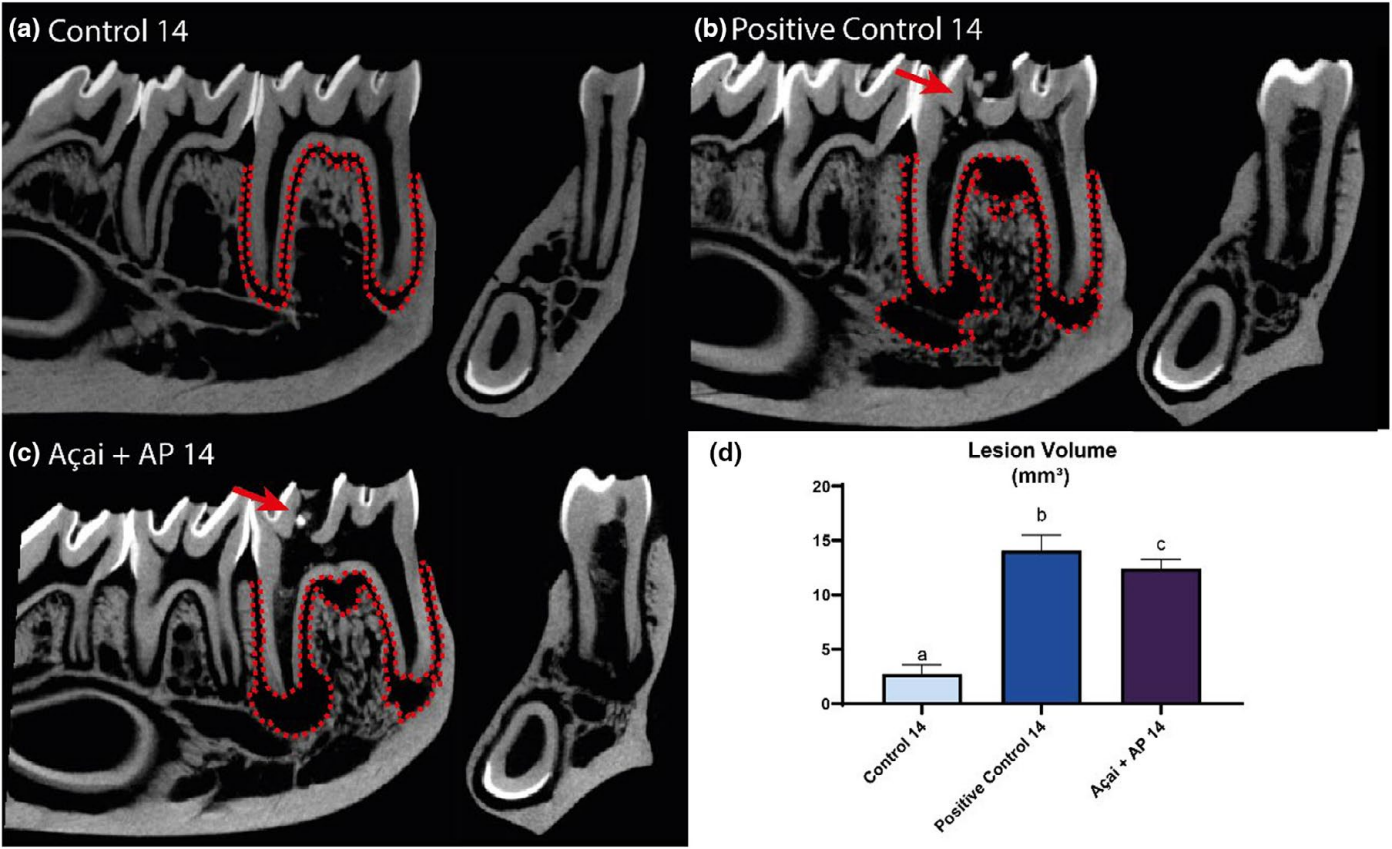
Evaluation of the periradicular area in rats exposed to experimental apical periodontitis with or without administration of clarified açai (0.01 mL/g/day for 14 days). (a–c) Sagittal and transaxial slices of the hemimandibles with a red dashed line illustrating the region of interest and a red arrow highlighting the access obtained in the distal fossa of the first molars. (d) Lesion volume results for the three experimental groups at 14 days are expressed as mean ± standard deviation. Different letters indicate a significant difference (*p* < .05).

Hemimandible analyses revealed that the AP14 and AP + Açai14 groups exhibited significantly larger lesion volumes than the Control 14 group (*p* < .05). Moreover, the AP14 exhibited a higher lesion volume than that of the AP + Açai14 group (Control 14: 2.74 ± 0.42; AP14: 14.07 ± 0.70; AP + Açai14: 12.40 ± 0.31; *p* < .05), as shown in Figure 4.

**FIGURE 5:**
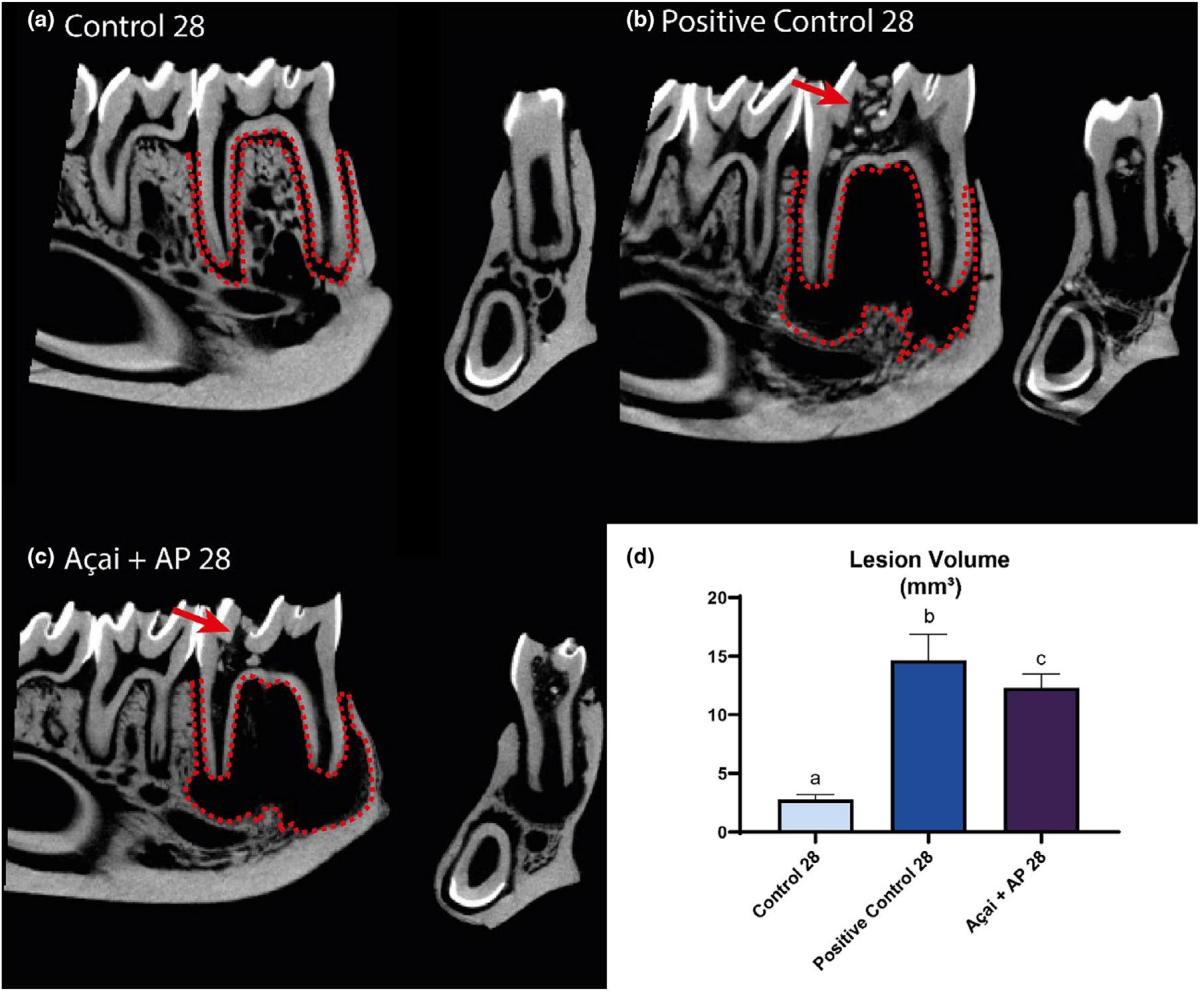
Evaluation of the periradicular area in rats exposed to experimental apical periodontitis with or without administration of clarified açai (0.01 mL/g/day for 28 days). (a–c) Sagittal and transaxial slices of the animal's hemimandibles with a red dashed line illustrating the region of interest and a red arrow highlighting the access made in the distal fossa of the first molars. (d) Lesion volume results for the three experimental groups at 28 days are expressed as mean ± standard deviation. Different letters indicate a significant difference. One‐way anova followed by Tukey's post hoc test, *p* < .05.

The results for the 28‐day animals followed the same pattern, with a higher volume in the AP28 and Açai+AP 28 groups than in the Control 28 group (*p* < .05). The Açai + AP28 group had a lower lesion volume (Control 28: 2.76 ± 0.0.21; AP28: 14.64 ± 1.12; AP + Açai28: 12.28 ± 0.42; *p* < .05) compared with that of the AP28 group, as shown in Figure 5.

#### The açai gavage altered alveolar bone quality/volume in the mandibles of rats

##### Fourteen‐day groups

The Control 14 group had the highest values of bone volume (Control 14: 6.01 ± 0.34; AP14: 4.02 ± 0.32; AP + Açai14: 4.80 ± 0.28) and BV/TV (Control 14: 44.81 ± 4.11; AP14: 21.61 ± 0.74; AP + Açai14: 29.89 ± 1.48) parameters, followed by the AP + Açai14 and AP14 groups (Control 14 > AP + Açai14 > AP14; *p* < .05).

The Control 14 group exhibited higher Tb.Th (Control 14: 0.182 ± 0.015; AP14: 0.115 ± 0.006; AP + Açai14: 0.129 ± 0.005) and Tb.N (Control 14: 2.593 ± 0.235; AP14: 1.99 ± 0.017; AP + Açai14: 2.098 ± 0.066) parameters (*p* < .05) than those of the AP14 and AP + Açai14 groups, which showed no differences between them (Control 14 > AP14 = AP + Açai14).

**FIGURE 6:**
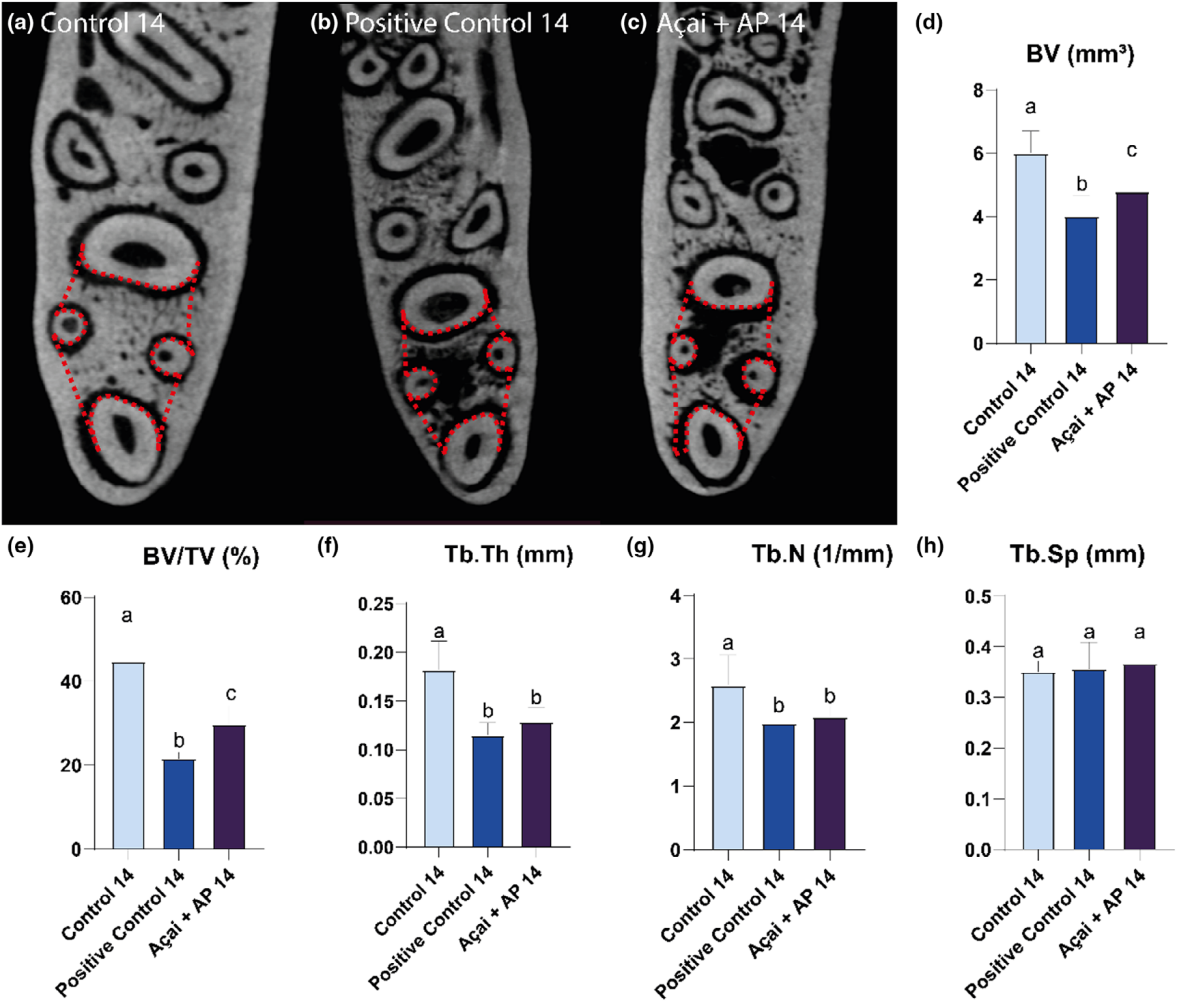
Evaluation of interradicular bone in rats exposed to experimental apical periodontitis with or without administration of clarified açai (0.01 mL/g/day for 14 days). (a–c) Coronal slices of the animal's hemimandibles with a red dashed line illustrating the region of interest. (d–h) Results of the bone parameters investigated for the three experimental groups after 14 days and expressed as mean ± standard deviation. Different letters indicate a significant difference. One‐way anova followed by Tukey's post hoc test, *p* < .05.

No significant differences were observed in Tb.Sp amongst the groups (Control 14: 0.351 ± 0.011; AP14: 0.356 ± 0.026; AP + Açai14: 0.368 ± 0.014; *p* > .05). The results are shown in Figure 6.

##### Twenty‐eight‐day groups

The Control 28 group had the highest BV (Control 28: 6.18 ± 0.17; AP28: 4.38 ± 0.20; AP + Açai28: 5.47 ± 0.15) and BV/TV (Control 28: 43.64 ± 1.41; AP28: 24.56 ± 1.68; AP + Açai28: 31.82 ± 1.70) values, followed by those of the AP + Açai28 and AP28 groups (Control 28 > AP + Açai28 > AP28; *p* < .05).

The Control 28 group exhibited higher values (*p* < .05) for the Tb.Th (Control 28: 0.191 ± 0.008; AP28: 0.121 ± 0.003; AP + Açai28: 0.141 ± 0.005) and Tb.N (Control 28: 2.277 ± 0.026; AP28: 1.913 ± 0.101; AP + Açai28: 1.890 ± 0.040) parameters compared to those of the AP28 and AP + Açai28 groups, with no significant differences between them (Control 28 > AP28 = AP + Açai28).

**FIGURE 7:**
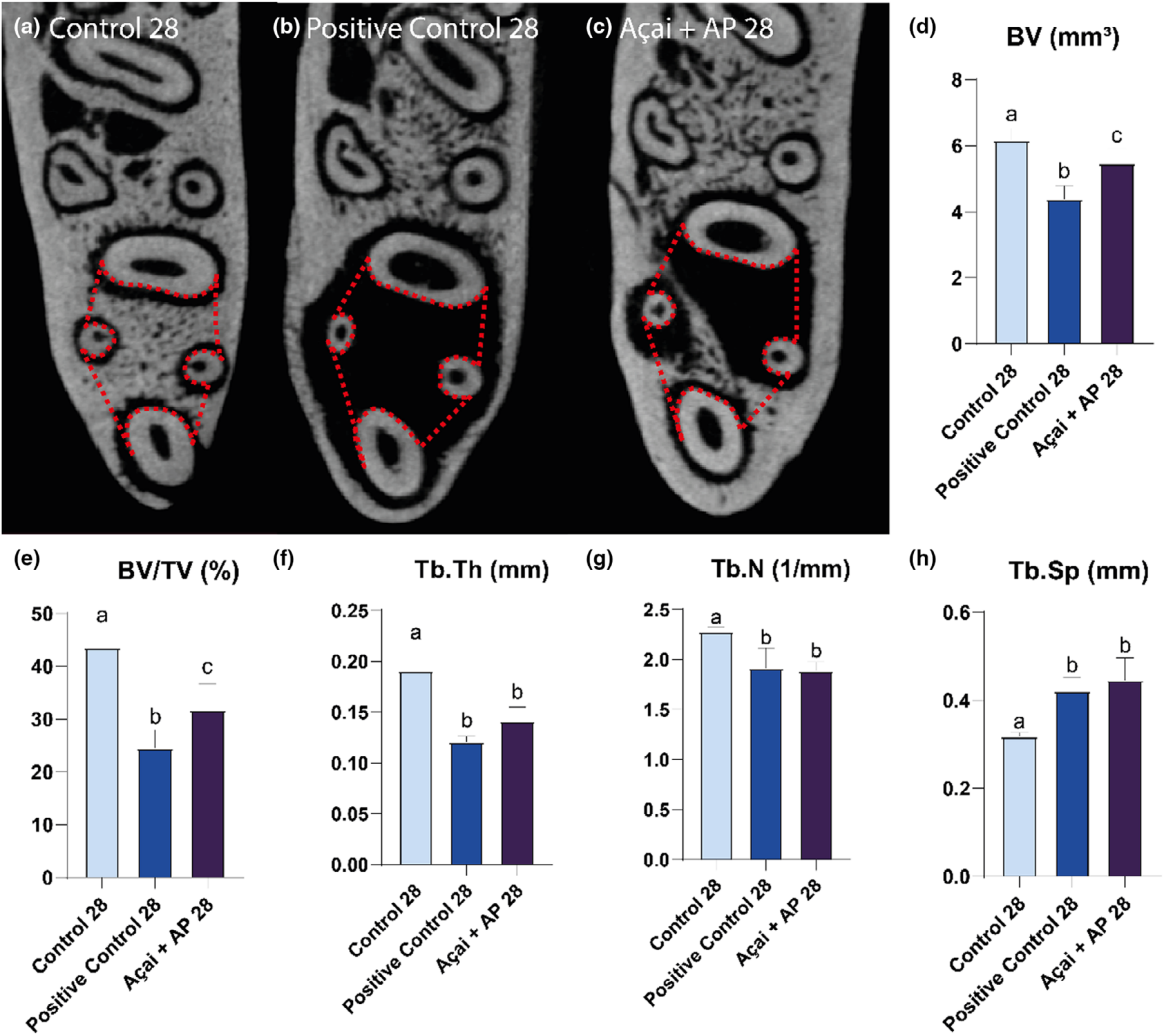
Evaluation of interradicular bone in rats exposed to experimental apical periodontitis with or without administration of clarified açai (0.01 mL/g/day for 28 days). (a–c) Coronal slices of the animal's hemimandibles with a red dashed line illustrating the region of interest. (d–h) Results of the bone parameters investigated for the three experimental groups after 28 days and expressed as mean ± standard deviation. Different letters indicate a significant difference. One‐way anova followed by Tukey's post hoc test, *p* < .05.

The Control 28 group had a lower value (*p* < .05) of Tb.Sp than that in the AP28 and AP + Açai28 groups, which showed no significant differences between them (Control 28: 0.318 ± 0.004; AP28: 0.422 ± 0.016; AP + Açai28: 0.446 ± 0.018). The results are shown in Figure 7.

### Oral açai administration did not promote histopathological modulation of periapical‐induced periodontitis

**FIGURE 8:**
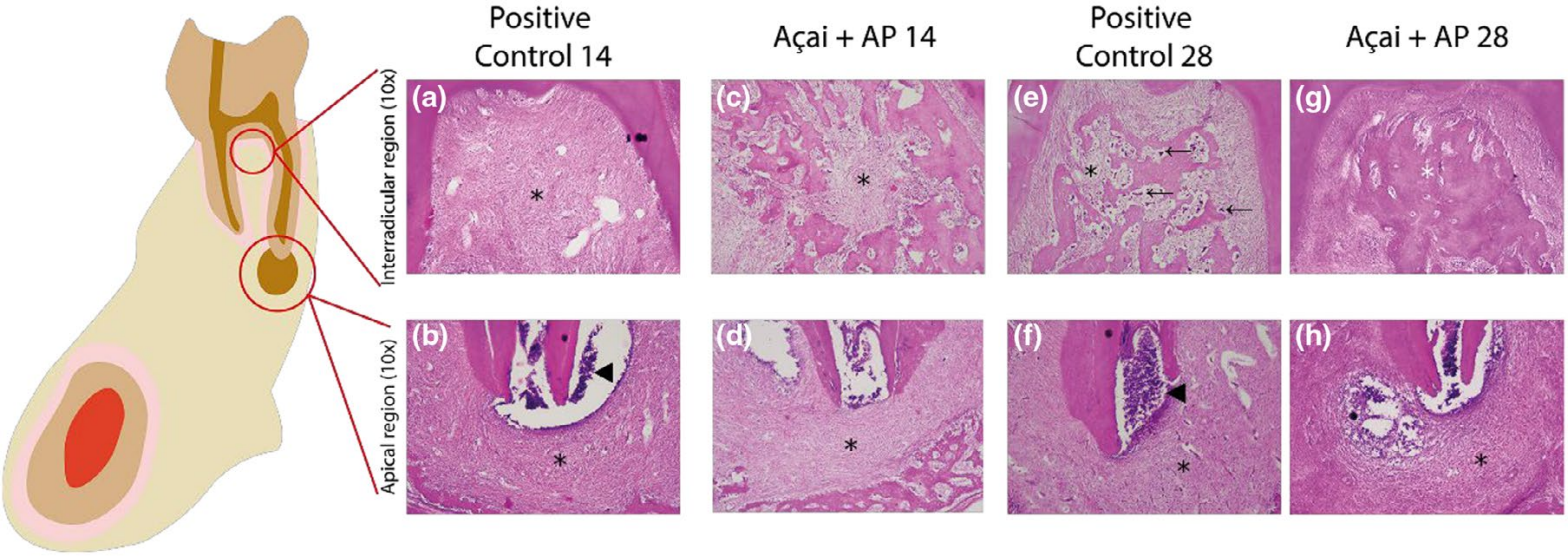
Results of the histopathological analysis. The descriptive analysis found that the 14‐day groups with apical periodontitis (Positive Control 14 and Açai + AP 14) displayed isolated areas of resorbed alveolar bone (as seen in images a and c) and a significant amount of inflammation in the periapical region (depicted in images b and d). The 28‐day groups with apical periodontitis (Positive Control 28 and Açai + AP 28) exhibited a greater level of inflammation (shown in images e and g) and the presence of bacterial colonies in the periapex (displayed in images f and h). Images (a, c, e, g) depict the interradicular region of all four groups. Images (b, d, f, h) show a close‐up view of the periapical area of each group at 10× magnification. In the images, the black asterisk indicates an area of bone resorption, the white asterisk indicates an area of preserved bone, the arrow indicates the presence of osteoclasts and the arrowhead marks a necrotic area with intense inflammatory infiltrate.

These tissue findings reinforced the microtomographic data. Both groups were monitored for 14 days and showed a moderate number of polymorphonuclear cells in the alveolar bone near the periapical region, which was consistent with the development of a pathological lesion. Figure 8a–d demonstrates the inflammation caused by these cells and the responses of the connective tissue of the periodontal ligament. The alveolar bone also showed signs of resorption, probably owing to an increased number of osteoclasts.

The 28‐day group showed a higher level of inflammation as indicated by the presence of bacterial colonies and a greater number of osteoclasts near the periapex. There were also instances of severe inflammation in specific areas of the interradicular region of the alveolar bone. Unlike the 14‐day group, the 28‐day group exhibited fewer osteoclasts and a long‐lasting periodontal ligament response. No difference was observed between the açai groups and the positive control groups. The results are presented in Figure 8.

### Daily consumption of açai modulated systemic oxidative biochemistry in the plasma of rats

**FIGURE 9:**
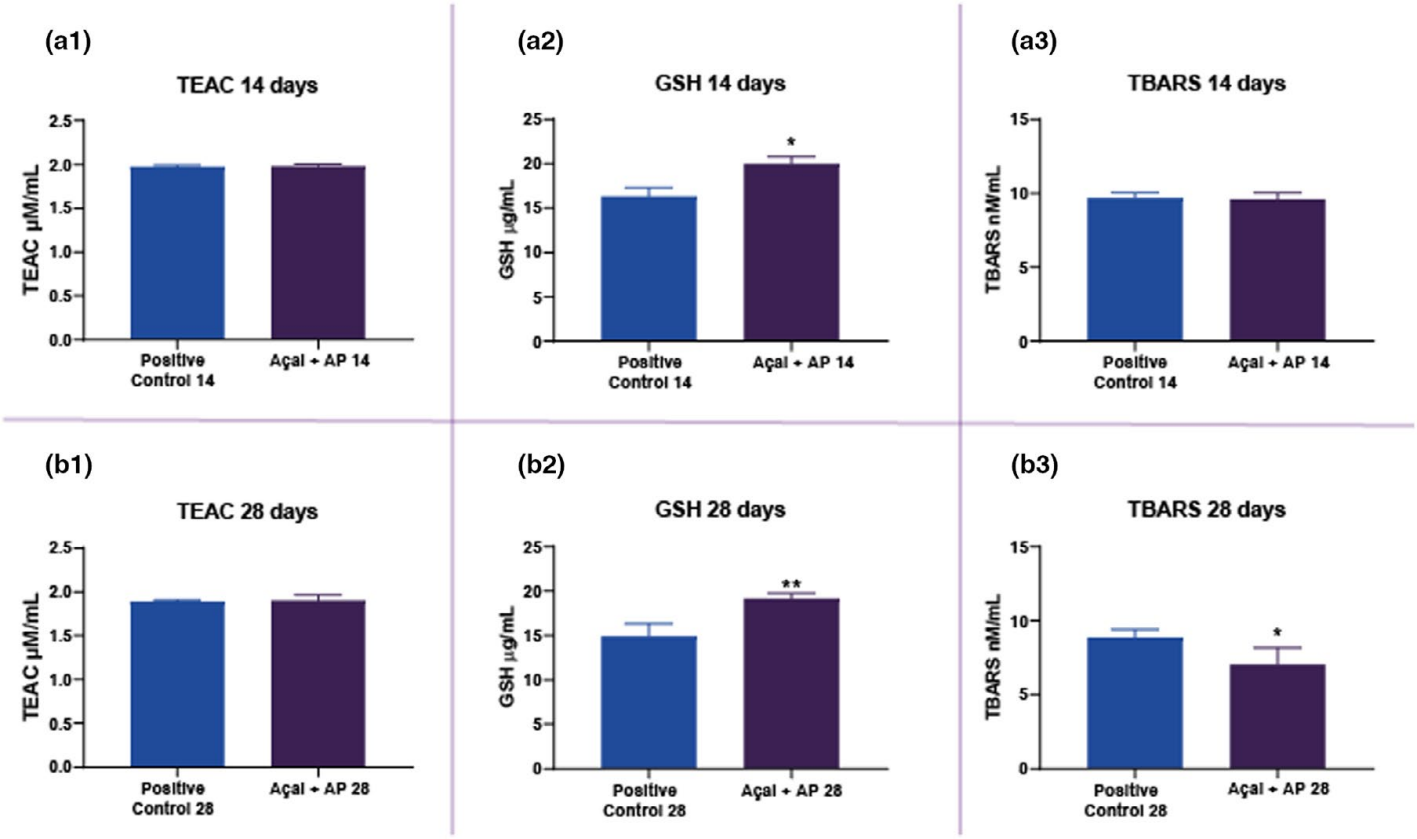
Oxidative biochemistry analyses. (a.1) Trolox equivalent antioxidant capacity (TEAC) analysis in 14‐day rats; (a.2) Reduced glutathione (GSH) analysis in 14‐day rats; (a.3) Thiobarbituric acid‐reactive substances (TBARS) analysis in 14‐day rats; (b.1) TEAC analysis in 28‐day rats; (b.2) Reduced GSH analysis in 28‐day rats; (b.3) TBARS analysis in 28‐day rats. Results are expressed as a percentage (%) of control (mean ± SEM). Different letters show a statistically significant difference (*p* < .05). Unpaired *t*‐test. “*” indicates *p* < 0.05 and “**” indicates *p* < 0.01. These symbols are automatically generated by the GraphPad Prism 9.0 software.

Regarding the TEAC analysis, no difference was observed between the groups (AP14: 1.975 ± 0.018; AP + Açai14: 1.981 ± 0.022; AP28: 1.89 ± 0.009; AP + Açai28: 1.903 ± 0.022; *p* > .05). Both the 14‐ and 28‐day animals of the AP + Açai groups showed higher glutathione (GSH) levels than those in their respective positive control groups (AP14: 16.350 ± 0.947; AP + Açai14: 20.01 ± 0.831; AP28: 14.957 ± 1.363; AP + Açai28: 19.15 ± 0.594; *p* < .05). Finally, the TBARS analysis showed no significant difference between the AP14 and AP + Açai14 groups (AP14: 9.69 ± 0.366; AP + Açai14: 9.59 ± 0.456; *p* > .05). However, the AP + Açai28 exhibited lower levels compared to those in the AP28 group (AP28: 8.873 ± 0.272; AP + Açai28: 7.067 ± 0.462; *p* < .05). All the biochemical results are shown in Figure 9.

## DISCUSSION

This study is the first to evaluate the effects of oral açai administration in a rat model of AP. Micro‐CT scan results showed that oral açai administration reduced the volume of AP lesions and improved bone quality, thereby slowing disease progression. Additionally, the study demonstrated that açai has a systemic antioxidant effect, with higher levels of GSH at both time‐points and lower levels of TBARS only after 28 days. Thus, the results of this study reject the null hypothesis.

To induce periradicular lesion formation, the teeth were left open in the oral environment until the end of the experiment. This widely used method has been described in previous studies that evaluated AP in rodents (Kakehashi et al., 1966; Kalatzis‐Sousa et al., 2017). However, methodological variations exist amongst previous studies, particularly regarding the duration of exposure, methods of pulp exposure and criteria for lesion confirmation. This lack of standardization was clearly demonstrated by Kalatzis‐Sousa et al. (2017), who highlighted that such variations might compromise the reproducibility of future studies on the topic. In our study, we followed a protocol similar to that of previous studies whilst ensuring lesion confirmation through both radiographic and histological analyses, reducing the potential impact of methodological differences.

Micro‐CT was selected to analyse the lesion size because it is more sensitive in detecting the AP than periapical radiography, which is more likely to miss AP even when it is still present (de Paula‐Silva et al., 2009). Furthermore, micro‐CT imaging is a rapid, reproducible and noninvasive method that provides results that are closely comparable with those obtained by histology and is useful for accurately quantifying changes in bone architecture in small biological specimens (Balto et al., 2000).

Clarified açai is a form of açai juice in which macronutrients, namely fibres, lipids and proteins, have been removed. The major phenolic compounds of açai were identified and quantified as cyanidin‐3‐glucoside (112.20 mg/L), cyanidin‐3‐rutinoside (543.30 mg/L), homoorientin (184.15 mg/L), orientin (144.81 mg/L), taxifolin deoxyhexose (13.06 mg/L), vitexin (10.57 mg/L) and isovitexin (10.18 mg/L). These compounds are commonly found in plants and collectively referred to as flavonoids. They have been the subject of extensive scientific research because of their potential health benefits and are found in fruits and vegetables, including açai drupes (Martins et al., 2018). Cyanidin‐3‐glucoside and cyanidin‐3‐rutinoside are anthocyanins, a category of flavonoids with strong antioxidant properties. Antioxidants play a crucial role in protecting cells from damage caused by harmful molecules known as free radicals (dos Santos et al., 2022). Homoorientin, orientin, taxifolin deoxyhexose, vitexin and isovitexin are members of the flavonoid family that have demonstrated various biological activities, including antioxidant, anti‐inflammatory and anticancer effects (Magalhães et al., 2020). These compounds are often studied for their potential health benefits, such as reducing the risk of chronic diseases, improving cardiovascular health and anti‐inflammatory effects.

The dosage used in this study (0.01 mL/g) was selected based on previous research that demonstrated the antioxidant properties and effects on pro‐inflammatory cytokines of açai (dos Santos et al., 2022; Souza‐Monteiro et al., 2019; Zhou et al., 2018). Our findings are consistent with these studies, as evidenced by the antioxidant parameters evaluated. Although the TEAC analysis did not show significant differences between the groups, the elevated GSH levels in both the 14 and 28‐day AP + Açai groups, compared to those in their respective positive control groups, suggest that oral açai administration positively modulates the antioxidant defence system. An increase in GSH, a crucial endogenous antioxidant, indicates an enhanced capacity to neutralize ROS and protect cellular integrity. Furthermore, the reduction in TBARS levels observed in the AP + Açai28 group compared to those in the AP28 group indicated a decrease in lipid peroxidation with prolonged oral açai administration, reinforcing the role of açai in mitigating oxidative damage. These observations support the hypothesis that açai, through its rich composition of phenolic compounds, exerts significant antioxidant effects that contribute to maintaining biochemical homeostasis.

The association between endodontics and general health cannot be ignored in clinical practice. It is common for lesions to remain untreated for long periods in individuals with AP, as symptoms are often absent (Cintra et al., 2021), resulting in a massive release of cells and inflammatory mediators (Jakovljevic et al., 2025; Matos‐Sousa et al., 2024), as well as increased local ROS production and oxidative stress over the years (Cintra et al., 2021; Georgiou et al., 2021). ROS modulate cell signalling and cause oxidant imbalances, which can result in long‐lasting oxidative stress that affects systemic health (Georgiou et al., 2021). This relationship between oxidative stress and AP is well established in the literature (Georgiou et al., 2021; Gomes et al., 2018; Hernández‐Ríos et al., 2017; Inchingolo et al., 2013; Vengerfeldt et al., 2017), demonstrating increased ROS levels and decreased antioxidant concentrations in subjects with AP, implying a systemic burden of AP. Several antioxidants such as vitamin E, flavonoids and other polyphenols have recently been exploited for their beneficial effects against oxidative stress (Pizzino et al., 2017), inflammation (Ricci et al., 2025) and bone resorption (Sales‐Junior et al., 2025). Phenolic compounds, the predominant phytochemicals in açai fruit (de Oliveira et al., 2021), have demonstrated several important systemic properties, including neuroprotective effects (Crespo‐López et al., 2019), tissue repair (Magalhães et al., 2021), antioxidant defence and reduction of oxidative stress (Pizzino et al., 2017), treatment/prevention of diseases such as diabetes, hypertension and hyperlipidemia (Magalhães et al., 2020), and inhibition of osteoclast activity (Brito et al., 2016). Açai is well known for its antioxidant properties (Crespo‐López et al., 2019; Souza‐Monteiro et al., 2019), capacity to inhibit osteoclasts (Brito et al., 2016) and ability to modulate alveolar bone loss (dos Santos et al., 2022). In the present study, anthocyanins were the major phenolic compounds. They exert beneficial effects on cardiovascular diseases by inhibiting inflammatory processes, endothelial dysfunction and antioxidant activities (Reis et al., 2016). Gavage with açai did not prevent the disease from occurring, as evidenced by the significantly smaller results in the control groups. However, the properties described above may explain why the volume of periapical lesions in the animals in the Açai group was smaller than that in the AP group at both time‐points studied.

Another important factor is that açai is a potential source of probiotics (Abe Sato et al., 2021). Probiotics can produce substances with antimicrobial properties. Additionally, some studies have suggested that probiotics may help boost the immune system, potentially enhancing the ability of the body to fight infections (Abe Sato et al., 2021). These factors may also explain the results reported in the referenced paper.

This study used two AP development times (14 and 28 days). Although directly comparing the results of the two different administration times was not an objective of this study, we used both time‐points based on a previous study by our group (Frazão et al., 2023) that showed differences in histopathological parameters between the two groups. On day 14, focal regions of moderate mononuclear inflammatory infiltrate were observed in the alveolar bone, whereas on day 28, an intense inflammatory infiltrate with bacterial colonies was observed. Micro‐CT analysis showed that the 28‐day group exhibited higher lesion volume, increased Tb.N and greater Tb.Sp. Studies have demonstrated that periapical lesions expand most rapidly between days 0 and 15, referred to as the “active phase,” after which they slowly enlarge until 20–30 days, known as the “chronic phase” (Minhoto et al., 2021; Wang & Stashenko, 1991). Histological analysis further supported these findings, with the 14‐day group displaying moderate mononuclear inflammatory infiltrates in the alveolar bone, indicating an ongoing immune response. Conversely, the 28‐day group exhibited more severe histopathological changes, including intense inflammatory infiltrates and the presence of bacterial colonies, suggesting progression to a more chronic inflammatory state. This difference in inflammatory response and tissue pathology between the two time‐points underscores the dynamic nature of AP progression and highlights the importance of disease duration in influencing both local and systemic pathologies. Based on the above observations, we aimed to understand the response to açai gavage in different stages of systemic and local damage. The results of this investigation are summarized below.

We previously investigated the effects of oral açai administration in a ligature‐induced periodontitis rodent model that showed the capacity to modulate alveolar bone loss, oxidative biochemistry and bone microstructure (dos Santos et al., 2022). The present results corroborate our previous findings, although these studies have focused on different pathologies, demonstrating a similar pattern of the effects of açai regarding AP and experimental periodontitis. When comparing both studies, açai minimized alveolar bone loss in both experimental periodontitis and AP. Regarding the BV/TV parameter, açai showed a similar pattern for both conditions, with higher values for the açai groups compared to the groups of animals with pathology. However, the results differed for Tb.Th and Tb.Sp. Although we did not assess inflammatory status, our other results suggest that açai can modify bone damage by modulating oxidative stress influencing inflammatory responses (dos Santos et al., 2022).

Several studies have reported the use of antioxidants for the treatment of AP in rodent models. Wolle et al. (2013) observed that the administration of Tempol for 21 days did not significantly reduce the extent of periapical lesions in rats with type 2 diabetes, as assessed radiologically, nor did it alter inflammation scores in histological assessments. Sarıtekin et al. (2019) reported that daily intraperitoneal injections of melatonin (10 mg/kg for 21 days) resulted in significantly smaller histomorphometric and radiographic areas of periapical bone loss. Aksoy et al. (2019) reported the inhibition of periapical bone loss using alpha‐lipoic acid (100 mg/kg for 28 days), and the treated group showed lower micro‐CT values for the surface area and volume of resorption cavities than the AP group. Brito et al. (2016) demonstrated that açai fruit extract can inhibit osteoclast differentiation and activity, leading to the hypothesis that açai gavage groups would exhibit lower lesion volumes. Notably, these studies differed in treatment duration, method of lesion volume evaluation and composition of the antioxidants used, with some applying treatment locally to the cells and others administering treatment systemically via gavage.

One of the limitations of our study is that multiple pathways may contribute to periodontal/periapical lesion progression (Cavalla et al., 2021), with oxidative stress being only one of them. However, AP leads to the local production of ROS at the root tip, regulates cellular signalling and leads to an imbalance of oxidants that causes pro‐inflammatory responses, metalloproteinase activation and AP lesion formation and progression. Biomarkers of oxidative stress are found in the blood and saliva of patients with AP. Additionally, the use of an animal model, whilst appropriate for preliminary mechanistic insights, may not fully reproduce the complexity of human endodontic pathologies (Ribeiro et al., 2025). Understanding the mechanisms of oxidative stress involved in chronic inflammation and the effects of antioxidants in patients with AP can provide important tools for improving local and systemic healing (Georgiou et al., 2021).

Our findings demonstrate a potential correlation between the intake of antioxidant‐rich foods and the progression of periapical lesions. This suggests that dietary antioxidants, such as those found in Açai, may play a significant role in modulating AP pathogenesis by mitigating oxidative stress. Clinically, these results open up new perspectives for incorporating antioxidant strategies as an adjunct to conventional endodontic therapies, potentially improving treatment outcomes and patient prognosis.

Furthermore, the study highlights the need for future research aimed at elucidating the molecular pathways that underpin this correlation. A deeper understanding of these mechanisms will not only validate the therapeutic potential of antioxidant interventions but also pave the way for translational clinical studies. Such investigations could ultimately lead to the development of novel, evidence‐based clinical protocols that integrate dietary modifications or supplementation as part of comprehensive endodontic treatment plans.

## CONCLUSION

In conclusion, our study clearly demonstrated that oral administration of clarified açai resulted in significantly reduced periapical lesion volumes and marked improvements in alveolar bone quality, as evidenced by the higher bone volume and BV/TV ratios detected by micro‐CT. Additionally, antioxidant analyses revealed that açai treatment significantly increased GSH levels at both 14 and 28 days and significantly reduced TBARS levels at 28 days, indicating a robust enhancement of the systemic antioxidant defence and a reduction in lipid peroxidation. These specific results provide compelling evidence that the antioxidant compounds in açai not only slow the progression of AP but also offer protective effects against both local and systemic oxidative damage. Further studies are warranted to elucidate the underlying molecular mechanisms and to evaluate the clinical applicability of these findings in endodontic therapy.

## AUTHOR CONTRIBUTIONS

Conceptualization: J.D.M.M., V.R.N.D.S., R.R.L. and P.A.R; methodology: L.O.B., P.F.S.M., J.M.M‐S., B.R.R.P. and J.M.P.; formal analysis: L.O.B., J.M.P., H.R. and R.R.L.; resources: F.M.C., P.F.S.M. and J.M.P.; data curation: V.R.N.D.S., L.O.B. and P.F.S.M.; writing original draft preparation: J.D.M.M., V.R.N.D.S., L.O.B. and B.R.R.P.; writing, review and editing: J.M.M‐S., F.M.C., H.R. and R.R.L.; visualization: J.M.M‐S., F.M.C., B.R.R.P. and H.R.; supervision: P.A.R and R.R.L.; project administration: P.A.R and R.R.L.; funding acquisition: R.R.L. All the authors have read and agreed to the published version of this manuscript.

## FUNDING INFORMATION

Rafael Rodrigues Lima is a researcher and member of the Conselho Nacional de Desenvolvimento Científico e Tecnológico (CNPq) and received a grant (grant number 312275/2021‐8). This research was funded by PROCAD Amazônia – CAPES (23038.005350/2018‐78).

## CONFLICT OF INTEREST STATEMENT

The authors declare no competing interests.

## Data Availability

All relevant data supporting the findings of this study are available in the manuscript. Additionally, any datasets generated or analysed during this study that were not included in the main text are available upon reasonable request from the corresponding authors. The materials described in the manuscript will be freely accessible to other researchers for non‐commercial purposes provided that participant confidentiality is maintained. Any restrictions regarding data sharing owing to privacy or ethical concerns are outlined in the manuscript.
